# Diagnosis and Treatment of Invasive Candidiasis

**DOI:** 10.3390/antibiotics11060718

**Published:** 2022-05-26

**Authors:** Natalia Barantsevich, Elena Barantsevich

**Affiliations:** Almazov National Madical Research Centre, Research Department of Microbiology and Nosocomial Indfections, Akkuratova, 2, 197341 Saint-Petersburg, Russia; natabara@mail.ru

**Keywords:** *Candida*, diagnostic tests, beta-D-glucan, T2Candida, mannan, procalcitonin, echinocandins, azoles, flucytosine, amphotericin B

## Abstract

*Candida* species, belonging to commensal microbial communities in humans, cause opportunistic infections in individuals with impaired immunity. Pathogens encountered in more than 90% cases of invasive candidiasis include *C. albicans*, *C. glabrata, C. krusei, C. tropicalis*, and *C. parapsilosis*. The most frequently diagnosed invasive infection is candidemia. About 50% of candidemia cases result in deep-seated infection due to hematogenous spread. The sensitivity of blood cultures in autopsy-proven invasive candidiasis ranges from 21% to 71%. Non-cultural methods (beta-D-glucan, T2Candida assays), especially beta-D-glucan in combination with procalcitonin, appear promising in the exclusion of invasive candidiasis with high sensitivity (98%) and negative predictive value (95%). There is currently a clear deficiency in approved sensitive and precise diagnostic techniques. Omics technologies seem promising, though require further development and study. Therapeutic options for invasive candidiasis are generally limited to four classes of systemic antifungals (polyenes, antimetabolite 5-fluorocytosine, azoles, echinocandins) with the two latter being highly effective and well-tolerated and hence the most widely used. Principles and methods of treatment are discussed in this review. The emergence of pan-drug-resistant *C. auris* strains indicates an insufficient choice of available medications. Further surveillance, alongside the development of diagnostic and therapeutic methods, is essential.

## 1. Introduction

*Candida* species are yeasts and members of the commensal microbial community in humans [1,2,3]. They are present on skin and mucous membranes of the oral cavity and gastrointestinal and genitourinary tracts [1,2,4,5,6,7,8,9]. These fungi cause superficial or invasive infections in individuals with impaired immunity [10,11,12]. The term invasive candidiasis refers to candidemia and infections of other normally sterile sites [13,14,15,16,17].

Risk factors for invasive *Candida* infections relate to a wide variety of conditions (hematologic and solid organ malignancies, burns, major surgery) and treatment methods (stem cell and organ transplantation, use of immunosuppressive agents, antibiotics, chemotherapy, hemodialysis, intravenous nutrition) [15,16,17,18,19,20,21,22]. Prolonged hospital stay and admission to intensive care units are also recognized as major risk factors for invasive candidiasis [23,24,25].

The genus *Candida* includes numerous species [16,26,27,28,29]. The most common species to form normal microbiota and potentially cause invasive infections are *C. albicans, C. glabrata, C. krusei, C. tropicalis*, and *C. parapsilosis*. These five species are responsible for more than 90% of invasive infections [16,30,31,32,33]. Other *Candida* spp. have also been reported worldwide as causative agents of invasive candidiasis in patients, but to a lesser extent [16]. A novel *Candida* species—*C. auris*—has recently emerged as an etiologic agent of invasive candidiasis worldwide [34,35]. The fungus first identified in 2009 in Japan was later found on all continents except Antarctica [36,37,38]. This species is closely related to the *C. haemulonii* complex and has five geographically and genetically distant clades [39,40]. The rise in cases occurred simultaneously in different regions [41,42]. *C. auris* easily forms biofilms, persist on different surfaces [37,43,44], and has high potential for nosocomial transmission [45,46].

Candidemia, an infection of the bloodstream with *Candida* spp., is the most frequently detected type of invasive *Candida* infection. It is the fourth leading cause of nosocomial bloodstream infections in the United States of America (USA) and the seventh in Europe [47]. The overall mortality rate of candidemia is 22–75% [48]. Attributable mortality is rarely estimated due to contributing severe underlying conditions, and ranges from 10% to 47% [27,49]. The prevalence of candidemia differs in various geographical regions, with 0.32/1000 admissions in South-Eastern China and up to 2.49/1000 admissions in Brazil [48]. The distribution of certain *Candida* spp. as the cause of invasive infections may depend on the underlying conditions and antifungal preparations used. *C. glabrata* is more likely to be isolated in patients with malignancy and transplantation, and *C. krusei* in patients with haematologic malignancies receiving fluconazole as prophylaxis [23].

The aim of the present study was to evaluate the diagnostic and treatment options for the management of invasive candidiasis.

## 2. Diagnosis of Invasive Candidiasis

### 2.1. Clinical Manifestations of Invasive Candidiasis

Clinical manifestations of invasive candidiasis are generally non-specific [50,51]. The few exceptions are specific lesions in chronic disseminated candidiasis (CDC) and ocular candidiasis. CDC is a form of invasive fungal infection affecting the liver, spleen and, rarely, other organs. It occurs most commonly in patients with acute leukemia treated with chemotherapy. The typical small, target-like abscesses in the liver or spleen, described as “bull’s-eyes”, and detectable on ultrasound, computed tomography or magnetic resonance imaging, accompanied by elevated levels of serum alkaline phosphatase, support diagnosis without additional mycological data. Ocular lesions are visible as progressive retinal exudates or vitreal opacities upon the ophthalmologic examination. Their diagnostic value increases if an episode of candidemia is present within the previous 2 weeks. *Candida* chorioretinitis or endophthalmitis occur in up to 45% of cases of invasive candidiasis associated with candidemia [52,53,54,55,56,57,58,59].

Other symptoms and signs of invasive candidiasis usually do not differ from infections of another origin [60]. This fact, along with often insufficient laboratory data, contributes to the complexity of differential diagnosis and leads to the introduction of the terms of possible, probable and proven invasive candidiasis [59,61,62,63]. These definitions are intended for epidemiological studies and the evaluation of diagnostic tests and antifungals, but not to guide individual patients’ care [59,63]. According to the last update of the regularly revised consensus on the diagnosis of invasive fungal infections, the definition of probable invasive candidiasis is based on the assessment of host factors, clinical manifestations, and mycological non-cultural evidence, while the term possible infection in connection with invasive candidiasis is no longer defined [59,61]. Proven invasive candidiasis usually requires confirmation with “gold standard” methods.

### 2.2. “Gold Standard” Methods for the Diagnosis of Invasive Candidiasis

The “gold standard” for the diagnosis of invasive candidiasis has long been positive cultures or, alternatively, histolopathology from normally sterile sites [49,64,65]. The last consensus guidelines on the diagnosis of invasive fungal infections introduced four possibilities to prove the diagnosis of invasive candidiasis. First, histopathologic, cytopathologic, or the direct microscopic detection of *Candida* pseudo- or true hyphae in specimens from normally sterile sites obtained by needle aspiration or biopsy. Second, positive culture from a sample obtained by a sterile procedure from a normally sterile site with clinical or radiological abnormality consistent with infection. This point includes samples from freshly placed drains (within 24 h). Third, the detection of *Candida* species by polymerase chain reaction (PCR) with subsequent DNA sequencing if yeasts are found microscopically in paraffin-embedded tissue. Fourth, blood culture positive for *Candida* species [59].

Candidemia, as the most frequently diagnosed invasive infection, results in deep-seated candidiasis in about 50% of cases due to hematogenous dissemination [66]. Cultures of *Candida* spp. become positive with the concentration of 1 CFU/mL, demonstrating high efficacy in discovering viable *Candida* cells [67]. The easiest test to diagnose invasive candidiasis is the blood culture test, though the efficiency of the procedure is low: *Candida* spp. are isolated from blood in only 21–71% of patients with autopsy-proven invasive candidiasis [68]. The sensitivity can be improved with an increase in the volume of the blood sample and the frequency of blood testing. These culture methods retain their importance, and will continue to do so in the years to come, due to the possibility of the isolation, identification and susceptibility testing of the infectious agent [68,69,70]. The main drawback of culture methods is that they are long, with a 72–96 h turnaround time leading to delays in proper treatment that results in increased mortality [67,71,72]. Another disadvantage is poor performance in neonates with candidemia and concurrent *Candida* meningitis when blood, as well as cerebrospinal fluid cultures, are generally sterile [27]. These facts have led to the rating of positive urine cultures, similar to blood ones, and the use of surrogate tests including thrombocytopenia and elevated C-reactive protein as predictors of candidemia in infants [73,74].

Samples are taken directly from the deep-seated sites of infection culture *Candida* in less than 50% of patients with invasive candidiasis: the possible reasons encountered are low concentration, uneven distribution of viable cells, and the small size of the sample [67]. The diagnostic struggle in deep-seated *Candida* infections is aggravated by invasive techniques required for sampling that are risky or contraindicated to patients with severe underlying conditions [67].

Special stainings, such as the periodic acid–Schiff stain, are capable of detecting polysaccarides and glycoproteins of the fungal cell wall; the Grocott–Gomori methenamine silver stain that targets carbohydrates is also used in histopathology [64,75,76,77,78,79]. The use of stains with fluorescent brighteners can increase sensitivity [27].

### 2.3. Identification of Candida Species

The identification of the etiologic agents of invasive candidiasis is a very important step in diagnostic procedures due to the high diversity of *Candida* species and the intrinsic or acquired resistance typical to selected species [80].

**Figure 1:**
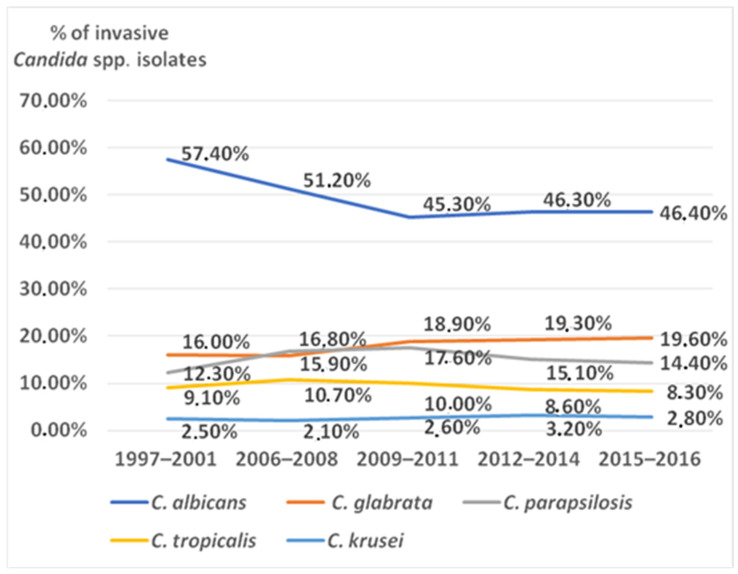
Candida species most frequently isolated in patients with invasive candidiasis during the SENTRY study.

During the Antifungal Surveillance Program of the SENTRY study, which was ongoing for 20 years from 1997 until 2016 in 135 medical centers in 39 countries, 20,788 invasive *Candida* isolates were collected. *C. albicans* was the most prevalent species: it accounted for 46.9% cases, followed by *C. glabrata* and *C. parapsylosis* with 18.7% and 15.9%, respectively. *C. tropicalis* was isolated in 9.3%, *C. krusei* in 2.8%, and miscellaneous *Candida* species in 6.5% of cases of invasive candidiasis (Figure 1) [81].

With the development of the modern taxonomy of microorganisms based on phylogeny, sequencing and Matrix-Assisted Laser Desorption–Ionization Time-Of-Flight (MALDI-TOF) mass spectrometry were introduced into laboratory practice, with the former cornerstone identification methods based on biochemical and physiological properties of different *Candida* species having lost their reliability. Biochemical tests based on chromogenic media still retain their value in diagnostics due to their ability to help differentiate mixed cultures, though they have limited capacity in the identification of uncommon *Candida* spp. [82,83,84,85,86]. Conventional methods show only 15–76% accurate identification [87] and are time consuming [80]. The biochemical methods implicated in automated identification systems cannot be considered reliable either: the VITEK 2 system demonstrates only 15.2% correct identifications of *C. guilliermondii*. This error might seriously mislead the administration of the correct treatment for the patient, since this species has reduced susceptibility to echinocandins due to its *FKS* gene polymorphism [88,89]. The API ID32C biochemical test system misidentifies *C. parapsilosis* and other *Candida* spp., for example, *C. sake*, which could also result in the failure of treatment, as these two species have different resistance patterns *C. sake*, contrary to *C. parapsilosis*, has decreased susceptibility to fluconazole and other triazoles [88]. Conventional methods often lead to *C. famata* misidentification [87,88], *C. auris* is identified with biochemical assays incorrectly in most cases [39,90,91].

The most reliable method that is currently used for the identification of fungi in routine laboratories is the Sanger sequencing of the ITS region and/or D1-D2 domain [92,93,94]. However, the ITS region seems to have limitations in differentiating closely related fungal species due to a lack of resolution and the presence of non-homologous copies of ITS in the genome [93]. Sequencing provides precise *Candida* species identification but is time-consuming and requires qualified staff [95,96,97]. The well-performing molecular method capable of correctly identifying *Candida* spp. is MALDI-TOF mass-spectrometry [98]. The technique is based on the comparison of the generated unique spectral fingerprints of the tested fungus with the database of mass spectra [60]. It provides accuracy, is comparable with sequencing, and is capable of identifying a *Candida* isolate to species level in less than 15 min [98,99]. This method is cost-effective, does not require expensive reagents or a highly qualified team, and has wide libraries which can be extended in-house [99,100,101]. The only drawback is the price of the equipment that makes it too expensive for routine laboratories in low-income regions [100,101].

There are several disadvantages of the diagnostic techniques based on the culture or histopathology of invasive candidiasis, including low sensitivity and the fact that sampling from sterile sites may be harmful to patients at risk of invasive candidiasis due to severe underlying conditions [59,102]. This information led to the study of *Candida* cultured from non-sterile sites in critically ill patients as an indicator of invasive candidiasis. Earlier studies demonstrated the association of *Candida* isolated from multiple non-sterile sites with candidemia, and further applied genetic techniques with confirmed strong associations of *Candida* strains derived from sterile and non-sterile samples [103,104,105]. This approach cannot be used to establish the diagnosis of invasive candidiasis, though it may be helpful in some cases without culture-proven infection as it allows the determination of the most probable infectious agent and the antifungal susceptibility testing of alternative isolates from probes other than normally sterile fluids or organs.

### 2.4. Non-Cultural Laboratory Techniques

The low sensitivity of “gold standard” methods has led to the development of non-cultural and non-histopathological laboratory techniques for the diagnosis of invasive candidiasis.

The first non-cultural diagnostic tools for invasive candidiasis were tests based on *Candida* antigens and anti-*Candida* antibody detection in patients’ serum [67]. Most *Candida* antigens demonstrated low concentrations and were rapidly cleared from the patients’ blood. The cell wall antigens mannan and β-D-glucan appeared to be promising targets [67,106,107]. The first tests to be introduced into practice were immune assays capable of detecting mannan and anti-mannan antibodies. The estimated sensitivity and specificity of both methods used simultaneously did not exceed 55% and 65%, respectively [108]. Interestingly, immunoglobulin G, especially in IgG_2_ assays, performed better than immunoglobulin M assays, and was able to suggest previous *Candida* infections in patients [67,109]. A high level of antibodies can be detected in patients with prior *Candida* infection without concurrent invasive candidiasis, especially in cases of autoimmune or other immunocompromised conditions, thus explaining the low positive predictive value of a single antibody detection test. The increase in antibody concentration might be more helpful in the diagnosis of candidiasis, but it requires at least two blood samples taken in two weeks, does not provide the early diagnosis of infection, and is not discriminative enough in the diagnosis of invasive candidiasis. The detection of mannan can be misleading due to the wide distribution of *Candida* spp. in numerous biotopes of the human body. Currently, neither mannan nor anti-mannan antibody detection have been approved for the diagnosis of invasive candidiasis by the US Food and Drug Administration (FDA).

The detection of beta-D-glucan, a cell wall compound of *Candida* spp., is used as a biomarker of invasive fungal infection with a sensitivity of 92% and specificity of 81% for the diagnosis of invasive candidiasis [108,110,111,112,113,114,115]. The main drawback of this method is the presence of beta-D-glucan in other fungi, including *Aspergillus* spp. and *Pneumocystis jirovecii*, that makes its use unreliable in the diagnosis of infections caused by *Candida* spp. Still, the high negative predictive value of this test is helpful in the exclusion of the diagnosis of invasive candidiasis. The test is approved by the FDA for the diagnosis of invasive fungal infections and is recommended as mycologic evidence for the diagnosis of probable invasive candidiasis (Table 1) [59,115].

The *Candida albicans* germ tube antibody test (CAGTA), intended to discriminate between infection and colonization, detects antibodies against antigens of the mycelium of the fungus in human serum or plasma. It demonstrates variable sensitivity (53–74%) and specificity (57–92%) with lower sensitivity for non-*albicans Candida* spp. [108,114,116,117]. The combination of various non-cultural tests—e.g., CAGTA, beta-D-glucan or mannan antigen tests—has a higher negative predictive value than single tests, and has been proved to be useful for decision making to discontinue unnecessary therapy in suspected invasive candidiasis [114]. The combination of two tests, including one of the *Candida* antigen detection methods and procalcitonin, seems to be even more promising in clinical practice when the early differential diagnosis of infection is crucial in severely ill patients. Procalcitonin levels in invasive candidiasis do not exceed 2 ng/mL opposite to highly elevated levels in bacterial, especially Gram-negative sepsis. The combination of procalcitonin level <2 ng/mL with the positive β-D-glucan test demonstrates sensitivity of 66% and specificity of 98% for invasive candidiasis [27,115].

PCR provides the identification of *Candida* spp. in 2–4 h and the possibility of monitoring the infection by indicating persistence or resolution [64]. This method showed high specificity and sensitivity in one trial 92% and 95%, respectively) [64]. PCR-based tests, which are highly effective in the diagnosis of infections caused by pathogenic bacteria or viruses, have lower reliability in the diagnosis of invasive candidiasis. The problem arises from the low number of *Candida* cells in the blood, often under 1 CFU/mL, and the wide distribution of fungus and its DNA in the human body and the environment, as well as similarity between human and fungal DNA [27,118,119]. Still, further development of PCR tests could increase the rate of detection of invasive candidiasis. There is already a wide variety of commercially available tests with PCRs alone or PCRs with subsequent electrospray ionization mass spectrometry, sequencing, or T2 nuclear magnetic resonance allowing the detection of *Candida* DNA directly from blood samples [120,121,122,123,124]. The latter, known as T2Candida panel (T2 Biosystems, Lexington, MA, USA), with sensitivity and specificity values of 91.1% and 99.4%, respectively, has been approved by the FDA and included in the list of non-cultural methods suitable for the diagnosis of probable invasive candidiasis and the detection of *C. albicans*, *C. glabrata*, *C. parapsilosis*, *C. tropicalis*, and *C. krusei* in whole blood (Table 1) [59,125,126].

Another non-cultural approach to the diagnosis of invasive candidiasis—the detection of *Candida* metabolites—has long been considered promising. The first metabolite to be used in the diagnosis was D-arabinitol, a metabolite produced by the several most common *Candida* spp. associated with invasive candidiasis, with the exception of *C. krusei* and *C. glabrata*. It can be measured in serum or urine [127]. This approach, however, did not confirm its effectiveness in the diagnosis of invasive candidiasis, and the detection methods were not standardized or validated [127].

With the development of omics technologies—genomics, transcriptomics, proteomics, and metabolomics—new hopes for the more precise diagnosis of invasive candidiasis have emerged. The study of the metabolomes of different *Candida* spp. seem to be currently the most promising and capable to define candidate compounds for approved diagnostic methods. Several databases of metabolomes of *Candida* spp. already exist (the YMBD or the Yeast Metabolome Database, the METLIN Metabolite and Chemical Entity Database, the LMISSD or LIPID MAPS In-Silico Structure Database, etc.) [128,129]. The work on the metabolomic profiles of different *Candida* spp. is currently at the early stage, and their potential for application in diagnostics is to be estimated in the future.

Genomic studies could provide the precise identification of infectious agents and antifungal susceptibility testing directly from a sample or an isolate. These methods are currently widely used in science and need trials to estimate their usefulness in routine practice. The possible implementation is additionally dependent on the decrease in the price of equipment and reagents, as well as the development of standardized procedures. Recent genomic studies in patients have revealed new host factors for invasive candidiasis: the significant association between candidemia and the single-nucleotide polymorphisms (SNPs) in *CD58, LCE4A*, and *TAGAP* loci has been demonstrated. The combination of two or more hazardous alleles resulted in a 19.4-fold increase in risk of candidemia [130,131]. The evaluation of host factors for invasive candidiasis with human genomics can improve diagnostics and estimate the patients in need of prophylaxis.

### 2.5. Antifungal Susceptibility Testing

The antifungal susceptibility of a *Candida* isolate is a cornerstone for the choice of antimicrobial agent and the treatment of invasive candidiasis. There are two major institutions issuing regularly updated guidelines for antifungal susceptibility testing performance and analysis: the Clinical and Laboratory Standards Institute (CLSI) and the European Union Committee on Antimicrobial Susceptibility Testing (EUCAST) [132,133]. The latter is easier to follow due to the free data provided. Both institutions issue well-established recommendations for testing performance in fungi, though they have insufficient information on some aspects of antifungal susceptibility analysis. EUCAST currently provides neither information on the assessment of the minimal inhibitory concentrations (MIC) of antifungals for *C. guillermondii*, nor caspofungin and isavuconazole interpretive guidelines for *Candida* spp. [133,134]. The emerged *C. auris*, which is capable to cause outbreaks of bloodstream infections in intensive care units, does not have any guidelines in either system. The only recommendations issued are tentative MIC breakpoints by the US Centers for disease control and prevention that lack information for all azoles except fluconazole and 5-fluorocytosine [135,136]. Another problem has arisen from the recently revealed inaccuracy of the Sensititre YeastOne panels widely used in routine laboratories, which have previously been considered precise tools for studying MIC in a wide variety of antifungal agents against *Candida* spp., predominantly for the assessment of MIC in echinocandins and fluconazole (i.e., in detecting caspofungin activity against *C. krusei* and *C. glabrata*, or fluconazole activity against *C. parapsilosis)* [137,138,139,140]. Vitek 2 can provide falsely high MICs of amphotericin B and caspofungin against *C. auris* [141,142,143]. The CLSI or EUCAST reference methods require high qualifications of the staff. As a rule, these tests are performed in reference laboratories, thus leaving patients with delayed answers; this is critical for the right choice of therapy and outcome [138,144,145]. The upgrade in interpretive criteria and the further development of precise and convenient test systems for everyday use could help improve patients’ care.

## 3. Treatment of Invasive Candidiasis

### 3.1. Principles of Therapy

The outcomes in patients with invasive candidiasis are generally dependent on mycological cure, the severity of the underlying conditions, and the time frame of therapy. The delay in the introduction of antifungal treatment for each 12–24 h may result in increases in mortality rate of up to 100% [49].

There are three major points that affect the outcome and duration of invasive candidiasis. First is the early diagnosis of infection. This requires the analysis of risk factors and clinical manifestations and the prompt use of all available approved cultural and non-cultural diagnostic methods [27,146]. Second is the search for the possible source of infection and its removal. It is very important to eliminate all blood and urine catheters. often encountered as sources of infection. as well as prosthetic devices where possible. All catheters and devices should be cultured upon removal. The surgical debridement of the site of infection has to be performed or, alternatively, the drainage of abscesses or infected peritoneal or pleural fluids should be performed [21,27,69,126,147]. Third, early effective systemic antifungal therapy has to be administered [69,126]. The delay in the administration of antifungal therapy, inappropriate formulations, and inadequate dosages result in higher mortality rates [148,149,150]. It is important to consider both the susceptibility of the yeast and drug–drug interactions along with the PK/PD data which have a high impact on certain loci of infections (e.g., in *Candida* endocarditis, *Candida* endophthalmitis, the central nervous system, or bone tissue involvement) [27].

The deficiency in reliable and sensitive methods for the diagnosis of invasive candidiasis and the long time (2–4 days) necessary for the isolation of fungus and susceptibility testing makes the early treatment that is crucial for a positive outcome challenging. The major aim is to start therapy as early as possible and use the effective regiment, but this is not easily feasible. There are several regularly updated detailed guidelines for the treatment of invasive candidiasis that can be helpful to healthcare professionals [150,151].

### 3.2. Antifungal Preparations

Systemic antifungals are used in the treatment of invasive candidiasis. There are four classes of preparations that differ in their mechanisms of action. One of the earliest systemic antifungal preparations introduced to clinical practice in 1959 was polyene amphotericin B [152]. It has been successfully used in the treatment of different invasive fungal infections, including candidiasis, since that time. Polyenes bind to ergosterol, the major component of the fungal cell membrane, creating pores and subsequent cell death [153,154]. These agents have a broad spectrum and a potent fungicidal effect. Most *Candida* species retain susceptibility to systemic polyenes. However, resistance is frequently detected in *C. lusitaniae* [155]. Amphotericin B and its lipid formulations are the only systemic polyenes available with lipid formulations less toxic and better tolerated by patients [156]. The use of conventional amphotericin B is limited by often-encountered individual intolerance and nephrotoxicity [157,158,159].

5-fluorocytosine (flucytosine) was developed as an antimetabolite in 1957. It inhibits fungal protein synthesis after being converted to 5-fluorouracil by cytosine deaminase and incorporated into fungal RNA, replacing uridylic acid. It is also a potent inhibitor of fungal DNA synthesis through the inhibition of thymidylate synthetase [160,161]. The drug is effective against a wide range of *Candida* spp., is well tolerated, and has a synergistic effect with amphotericin B. Flucytosine is used in combination with amphotericin B in invasive candidiasis in neonates with *Candida* meningitis due to its superb penetration into the cerebrospinal fluid. The use of flucytosine monotherapy is limited due to easily emerging resistance [162,163,164,165]. Hematologic and hepatic toxicities are associated with flucytosine administration. The careful monitoring of blood cell count is recommended. The adjustment of dose is advocated in patients with renal disfunction: serum concentration monitoring is helpful [164].

Systemic azoles and currently widely used triasoles have several antifungal formulations, including fluconazole, itraconazole, voriconazole, posaconazole, ravuconazole, and isavuconazole. Azoles inhibit lanosterol 14α demethylase, a key enzyme of ergosterol biosynthesis. Azoles generally demonstrate fungistatic activity against *Candida* spp. Most of them are active against fungi, causing invasive candidiasis with some agents, and demonstrating decreased activity against certain species. Resistance to fluconazole is most common in *C. auris*, *C. glabrata*, and *C. parapsilosis*; *C. krusei* has intrinsic resistance to fluconazole. Resistance to other azoles is rarely encountered, though increasing in frequency [166,167,168,169].

Echinocandins (micafungin, anidulafungin, caspofungin) present the newest class of antifungals and feature a fungicidal effect in *Candida* species. Their mechanism of action is the inhibition of β-D-glucan synthase: the important enzyme in cell wall synthesis. All echinocandins have high efficiency in invasive infections and an exceptional safety profile. A novel echinocandin rezafungin with once-weekly dosing and activity against *Candida* spp., including subsets of echinocandin-resistant *Candida auris*, has been recently developed and is currently in phase III trials [170,171,172,173,174,175,176]. Echinocandins demonstrate wide distribution in different organs and tissues, except the brain and eyes [52,177].

### 3.3. Choice of Antifungal Preparations and Duration of Treatment

Early diagnosis and early efficient initial therapy play a crucial role in the outcome of infection: a 24 h delay in obtaining positive blood cultures is associated with an almost two-fold increase in mortality in cancer patients [178]. Echinocandins are considered the drugs of choice for initial therapy in most cases of invasive candidiasis [179,180,181], though it depends on the severity of infection, data on the effectiveness of the antifungal treatment in previous episodes and intolerance to antifungals, the involvement of organs that demand specific permeability, and the tissue distribution of the agent (central nervous system, valves, bones, articular tissues, etc). Data on the dominant pathogen and its antifungal susceptibility in certain hospital settings, such as wards and departments, especially in non-neutropenic patients when an exogenous source of infection is suspected, are also important (Table 2).

Echinocandins were effective as initial treatment in 70–75% of patients with invasive candidiasis, according to randomized clinical trials [27,180]. Lower than expected survival rate might be due to the insusceptibility of the fungus to the agent used, or other circumstances (severe underlying condition or infection, delay in diagnosis or the initiation of therapy, non-performed debridement, or poor permeability of the drug into the infected site) [182,183,184,185,186]. An analysis of seven randomized clinical trials in roughly 2000 patients with invasive candidiasis showed that choice of an echinocandin as the initial treatment was associated with a significantly lower 30-day mortality rate compared with azoles or amphotericin B [150]. Worse outcomes were associated with *C. tropicalis* infections, higher APACHE II score, and older age [150]. *C. parapsilosis* usually demonstrates higher MIC to echinocandins than other *Candida* spp. Nevertheless, data from several clinical trials have shown that initial therapy with any echinocandin is appropriate for patients with *C. parapsilosis* infection, contrary to infections caused by other *Candida* spp. with acquired resistance to echinocandins [187,188,189]. Two randomized trials compared an echinocandin with an azole as a first-line therapy of candidaemia. One of them demonstrated that overall response rates were lower with fluconazole (60%) examined against anidulafungin (76%); another provided similar data for isavuconazole (60%) compared with caspofungin (71%) [182].

De-escalation or step-down therapy with changes from echinocandins to azoles provides favorable results in invasive candidiasis with different organ involvement (Table 2). The step-down therapy is normally started after 3–7 days of initial treatment and is administered in accordance with the results of susceptibility testing. Fluconazole could be the perfect choice for infections caused by susceptible strains with high efficiency, well-tolerability, and oral formulations with absorption from the gastrointestinal tract above 90% (Table 2). Several clinical trials did not reveal any differences in the 30-day survival or mycological cure rates of patients who received echinocandin therapy and those who were transferred to de-escalation therapy with oral azoles, according to susceptibility testing after 5 days of treatment with echinocandin [184,190]. Another option for step-down therapy with azoles in invasive candidiasis is voriconazole active against *C. guilliermondii*, *C. glabrata* and *C. krusei* with reduced susceptibility or resistance to fluconazole. De-escalation therapy significantly decreases the financial burden of invasive candidiasis for healthcare systems, with reduced hospital costs and shortened stay, as azoles, unlike echinocandins, can be successfully administered as oral formulations.

Azoles have some advantages compared to echinocandins as a first-line therapy treatment for the involvement of certain organs. Contrary to echinocandins, azoles easily cross the blood–brain barrier, making them the drug of choice for initial treatment in cases with central nervous system involvement. Fluconazole is widely used in low-income countries with a low prevalence of azole resistance as the first therapeutic choice in all types of invasive *Candida* infections. Patients with invasive candidiasis and no previous exposure to azoles may receive azoles as initial therapy if they are stable hemodynamically and do not have an increased risk of *C. glabrata* infection. The group of patients at high risk of *C. glabrata* includes patients with cancer or diabetes mellitus, and the elderly [69]. There are trials of newer azoles (posaconazole, ravuconazole, isavuconazole) demonstrating excellent in vitro activity against *C. krusei*, *C. guilliermondii* and *C. glabrata*. They might be alternatives to echinocandins as a first-line therapy [27,191].

Amphotericin B deoxycholate or, preferably, lipid formulations may be considered in infections refractory to other systemic antifungals in *Candida* endocarditis or *Candida* endophthalmitis. Intolerance of other classes of antifungals in individuals may also favor the use of amphotericin B lipid formulations. Multidrug-resistant strains with resistance to azoles and echinocandins are encountered with increasing frequency in *C. glabrata* and *C. auris*, with amphotericin B lipid formulations successfully used in these cases. Pan-drug-resistant strains *of C. auris* with no available treatment options have already been reported [192].

The recommended duration of systemic antifungal therapy for candidemia is at least 14 days after the eradication of *Candida* spp. from the blood and the resolution of all symptoms and signs of infection [69,151]. In deep-seated invasive candidiasis, e.g., chronic hepatosplenic candidiasis or intra-abdominal candidiasis, the duration of treatment normally differs from several weeks to 6–12 months and is individually adjusted. It is guided by the rate of lesion resolution, physicians’ personal experience, and scarce and typically non-randomized studies [27].

## 4. Conclusions

Invasive candidiasis is a major course of morbidity and mortality with an incidence of up to 2.49/1000 admissions and an estimated attributable mortality for candidemia of 10–47% [27,33,34,49]. Five *Candida* species (*C. albicans*, *C. glabrata*, *C. parapsilosis*, *C. krusei*, *C. tropicalis*) are responsible for more than 90% of infections [16,30,31,32]. The diagnosis of deep-seated organ involvement is challenging, and blood cultures provide positive results in less than 40% of patients without concurrent candidemia [67]. Several non-cultural techniques have been developed in recent years that act as valuable diagnostic tools and are especially useful in excluding the diagnosis of invasive candidiasis due to a high negative predictive value. The combination of two tests—beta-D-glucan and procalcitonin—is highly recommended [27,115]. There is a clear demand for future research and the development of sensitive and precise diagnostic tools. The most promising seem to be omics technologies capable of providing new substances to be applied as valuable diagnostic tests for identification and susceptibility testing in *Candida* spp., as well as to determine host factors predisposing for infection. There are only four classes of systemic antifungals with echinocandins as the first line, and the use of azoles for de-escalation therapy is most widely adopted [27,180,184,190]. The further search for new classes of antifungals is essential taking into account the potential multi-drug resistance in *C. glabrata*, demonstrating an increasing frequency of resistance to echinocandins and azoles. Moreover, *C. krusei* has an intrinsic fluconazole resistance, combined with a reported decreased susceptibility to amphotericin B and flucytosine, and pan-drug resistance is emerging in *C. auris* [29,193,194,195,196,197,198,199].

## Figures and Tables

**Table 1:** Tests approved for the diagnosis of invasive candidiasis.

Test	Turnaround Time	Diagnostic Value	Sensitivity	Specificity	Notes
Culture	2–4 days	Positive	21–71%	N/A	Allows susceptibility testing
T2Candida	3–5 h	Positive	91%	99%	Approved for the detection of *C. albicans*, *C. krusei*, *C. tropicalis*, *C. parapsilosis*, and *C. glabrata* in whole blood.
β-D-glucan (Fungitell)	1 h	≥80 ng/L	92%	81%	Can be positive in other fungal infections
β-D-glucan + procalcitonin	1 h	≥80 ng/L <0.2 ng/mL	96%	98%	Can be positive in other fungal infections

**Table 2:** Therapy of invasive candidiasis and preferred medications.

Etiologic Agent of Invasive Candidiasis Therapy	*C. albicans*, *C. parapsilosis*, *C. tropicalis*	*C. krusei*, *C. glabrata*	*C. auris*
First-line therapy *	Echinocandin	Echinocandin	Echinocandin
Alternative first-line therapy	Fluconazole	Amphotericin B lipid formulations	Amphotericin B lipid formulations
Step-down therapy **	Fluconazole	Voriconazole	Susceptibility data required

Notes: * Liposomal amphotericin B and flucytosine are used in the central nervous system or for eye infections. ** Step-down therapy is based on MIC assessment in individual cases.
